# Deep annotation of long noncoding RNAs by assembling RNA-seq and small RNA-seq data

**DOI:** 10.1016/j.jbc.2023.105130

**Published:** 2023-08-04

**Authors:** Jiaming Zhang, Weibo Hou, Qi Zhao, Songling Xiao, Hongye Linghu, Lixin Zhang, Jiawei Du, Hongdi Cui, Xu Yang, Shukuan Ling, Jianzhong Su, Qingran Kong

**Affiliations:** 1 Oujiang Laboratory, Zhejiang Provincial Key Laboratory of Medical Genetics, Key Laboratory of Laboratory Medicine, Ministry of Education, School of Laboratory Medicine and Life Sciences, Wenzhou Medical University, Wenzhou, Zhejiang Province, China; 2 Oujiang Laboratory, Zhejiang Lab for Regenerative Medicine, Vision and Brain Health, Wenzhou Medical University, Wenzhou, Zhejiang Province, China

**Keywords:** transcriptome annotation, full-length transcript, ERV-lncRNA, mouse embryo development, iPSC

## Abstract

Long noncoding RNAs (lncRNAs) are increasingly being recognized as modulators in various biological processes. However, due to their low expression, their systematic characterization is difficult to determine. Here, we performed transcript annotation by a newly developed computational pipeline, termed RNA-seq and small RNA-seq combined strategy (RSCS), in a wide variety of cellular contexts. Thousands of high-confidence potential novel transcripts were identified by the RSCS, and the reliability of the transcriptome was verified by analysis of transcript structure, base composition, and sequence complexity. Evidenced by the length comparison, the frequency of the core promoter and the polyadenylation signal motifs, and the locations of transcription start and end sites, the transcripts appear to be full length. Furthermore, taking advantage of our strategy, we identified a large number of endogenous retrovirus-associated lncRNAs, and a novel endogenous retrovirus–lncRNA that was functionally involved in control of *Yap1* expression and essential for early embryogenesis was identified. In summary, the RSCS can generate a more complete and precise transcriptome, and our findings greatly expanded the transcriptome annotation for the mammalian community.

Long noncoding RNAs (LncRNA), a class of longer than 200 nts and nontranslated endogenous cellular transcripts, have been considered as by-products of transcription without functions. However, recently, they have emerged as new and fundamental transcriptional and posttranscriptional regulators (1, 2, 3, 4, 5, 6). Considering the versatility of the molecule *via* different modes of action and with different target specificity, the annotation and understanding of lncRNAs in various biological process are desirable. In humans and mice, up to 30% of transcription start sites (TSSs) are located in transposable elements (TEs), and more than two-thirds of lncRNAs incorporate TEs (7, 8, 9, 10). TE-associated lncRNAs exhibit clear tissue-specific and developmental stage–restricted expression patterns (11). In addition, endogenous retrovirus (ERV) long-terminal repeat (LTR) sequences with binding sites for pluripotency-specific transcription factors are enriched in stem cell-specific lncRNAs (12, 13); for example, the human pluripotency–associated transcripts are typically incorporated with HERV-H and contribute to formation of the blastocyst inner cell mass (ICM) and the maintenance of pluripotency (14). These findings point to a likely role for TE-associated lncRNAs in pluripotency and lineage commitment. However, the identities of specific lncRNAs are difficult to evaluate because these contain TEs and are found at very low–expression levels.

Among the sequencing-based techniques used to annotate RNA transcripts, next-generation RNA-seq is commonly utilized (15, 16, 17, 18, 19). However, because this strategy is unable to capture the ends of a given gene (20, 21), particularly those found at low expression levels. Although Alexandre Fort *et al.*(22) identified a class of stem cell-specific transcripts derived from long-terminal repeat, named nonannotated stem transcripts, using cap analysis of gene expression (CAGE), and transcript assemblies can be obtained based on data from CAGEscan (nanoCAGE combined with paired-end sequencing) and RNA-seq, the application is limited due to the large quantities of purified RNA that need to be used and the technical difficulty of CAGE (23, 24, 25). Long-read sequencing technologies can generate long sequences of kilobase-sized sequencing reads, and the recently developed single-molecule real-time sequencing on the Pacific Biosciences platform provides an accurate and sensitive tool for annotating full-length RNA transcripts (26, 27, 28). However, this platform is not accessible to most users, who thus have to rely on commercial sequencing services and standard sequencing facilities (29, 30). Therefore, there remain problems that cannot be addressed with the existing approaches.

Diverse RNA species are transcribed from regulatory elements and genes. Transcription factors and coactivators recruit Pol II to enhancer and promoter elements, where short (20–400 bp) RNAs are produced before the phosphorylation of the C-terminal domain of Pol II (31, 32). And, a number of small RNAs derived from the 5′ and 3′ UTRs of mRNAs have been identified in *Drosophila* (33, 34), HeLa cells (35, 36), T cells, and embryonic stem cells (37, 38). These studies raise the exciting possibility that small RNAs might help to determine the 5′ and 3′ terminals of transcripts. In this study, we developed RNA-seq combined strategy (RSCS) to perform transcript annotation in a wide variety of cellular contexts. This new method appears to annotate full-length RNA transcripts and can generate complete and precise transcriptomes. Furthermore, novel ERV-lncRNAs that play prominent roles in the control of gene expression programs were characterized. We demonstrate that the RSCS is valuable for annotating comprehensive transcript sets.

## Results

### RSCS annotation of transcriptome in mouse early embryos

**Figure 1:**
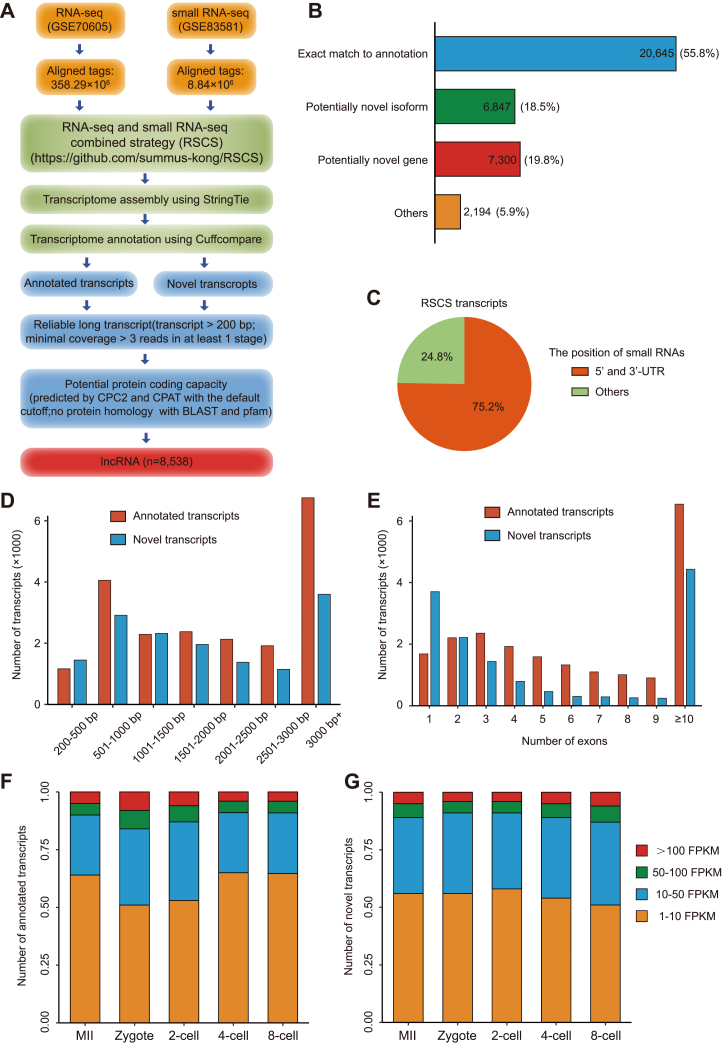
**Identification of the RSCS transcripts in mouse early embryos.***A*, bioinformatics pipeline of the RSCS for transcripts annotation. See “Experimental procedures” for details. *B*, annotation of the RSCS transcripts that were the combination of transcripts identified in the early embryos. The numbers and percentages of the RSCS transcripts are presented. *C*, *pie chart* showing the classification of RSCS transcripts based on the position of small RNAs mapping. The percentages of each category are shown. *D* and *E*, the length (*D*) and the exon count (*E*) distributions of annotated and novel transcripts identified by the RSCS. *F* and *G*, the expression of transcripts identified by the RSCS in the early embryos. The *bar plot* presents the percentage of annotated (*F*) and novel (*G*) transcripts classified by FPKM. FPKM, fragments per kilobase of exon per million mapped fragments; RSCS, RNA-seq combined strategy.

Examining small RNAs genome-wide distribution based on small RNA-seq data from mouse early embryos, we found more tags mapped to 5′ UTRs and 3′ UTRs of coding genes, compared to coding exons and introns (Fig. S1*A*). The substantial number of the UTR molecules and the molecules correlated with exons suggest that the small RNAs may help to annotate transcripts. To test this possibility, a computational pipeline, RSCS, was developed to assemble RNA-seq and small RNA-seq data (Fig. 1*A*). In this study, we profiled the transcriptome using public RNA-seq and small RNA-seq data (GSE70605, GSE83581) from mouse MII oocyte and zygote to eight-cell embryos by the RSCS. In total, 36,986 high-quality nonredundant transcripts were identified (Fig. 1*B* and Table S2). Importantly, the RSCS, rather than assembling double RNA-seq data, generated more potential novel transcripts, verifying the contribution of small RNA in transcript annotation (Fig. S1*B*). The small RNA tags were assembled to the terminals of most transcripts (Fig. 1*C*), and microRNA constitute over 80% of the terminal small RNA tags (Fig. S1, *C* and *D*). To identify and characterize the transcripts annotated by the RSCS, we compared the RSCS transcripts with the GENCODE annotation (vM20). The proportion of RSCS transcripts that were exactly matched to the annotation was 55.8% (n = 20,645) (Fig. 1*B*). Novel transcripts, including potentially novel genes (no overlapping with the reference annotation) and potentially novel isoforms, accounted for 18.5% (n = 68,47) and 19.8% (n = 7300) of the RSCS transcripts (Fig. 1*B*). And, the length and exon number of the novel transcripts presented a similar distribution to the annotated transcripts, validating the reliability of these novel transcripts (Fig. 1, *D* and *E*). Furthermore, we quantified the expression levels of RSCS transcripts in all the five stages. In each stage, we analyzed that those transcripts were identified with fragments per kilobase of exon per million mapped fragments (FPKM) >1, and nearly half of the novel and annotated transcripts were highly expressed (FPKM >10) in all the stages (Fig. 1, *F* and *G*). These results indicate that most of the annotated and novel transcripts identified by the RSCS are abundantly expressed in mouse early embryos.

### Validation of the RSCS transcripts

**Figure 2:**
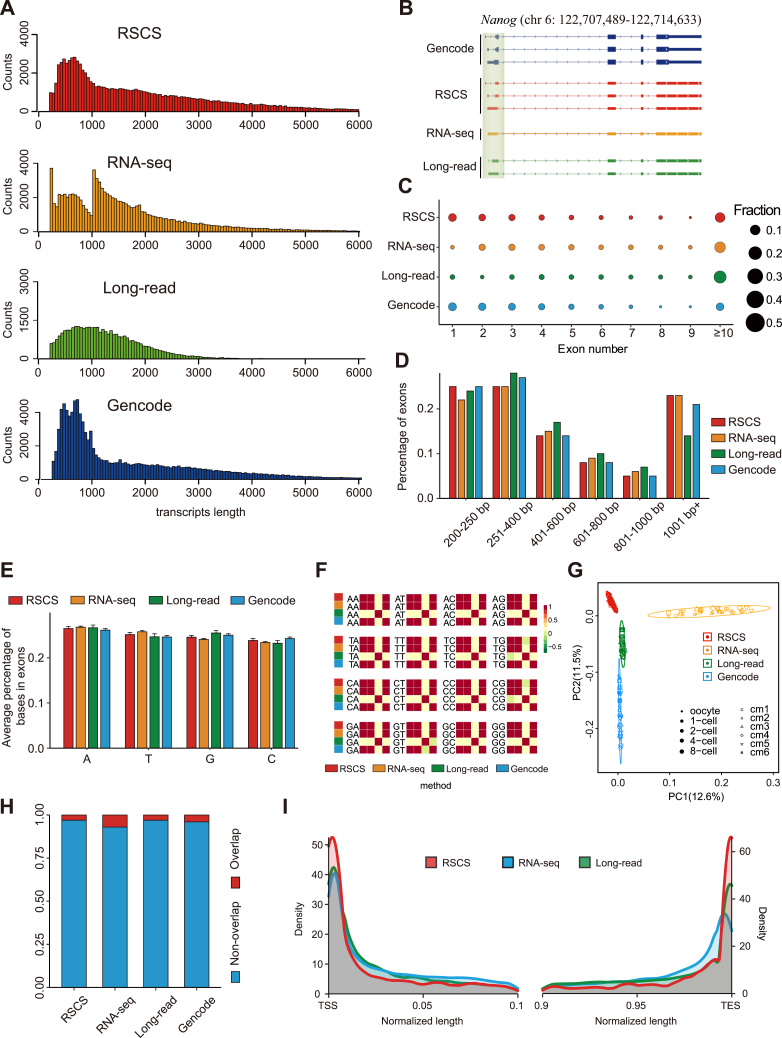
**Validation of transcripts identified by the RSCS.***A*, comparison of the length distributions of the transcripts identified by the RSCS (*red*), RNA-seq (*yellow*), and long-read data (*green*) with that detailed in mouse GENCODE (M26; *blue*). *B*, representative examples of *Nanog* isoforms annotated using the RSCS and other methods. *C* and *D*, the exon count (*C*) and length (*D*) of the transcripts identified by the RSCS, RNA-seq, long-read data, and those detailed in mouse GENCODE (M26). *E*, average percentage of bases in exons of the transcripts identified by the RSCS, RNA-seq, long-read data, and that detailed in mouse GENCODE (M26). *F*, heatmap showing Pearson correlation coefficients based on the motifs in exons of the transcripts identified by the RSCS, RNA-seq, long-read data, and those detailed in mouse GENCODE (M26). Each dinucleotide is plotted in a 4 × 4 matrix, and different methods are marked by different colors, and the bar shows the correlation of dinucleotide ratios in transcripts. *G*, PCA of the complexity of Markov model values in exons of the transcripts identified by the RSCS, RNA-seq, long-read data, and those detailed in mouse GENCODE (M26). *H*, *bar plot* represents the percentage of low-complexity regions obtained from RepeatMasker in exons of the transcripts identified by the RSCS, RNA-seq, long-read data, and that detailed in mouse GENCODE (M26). *I*, Kernel density estimates of the distribution of the TSS and TES localizations of the transcripts identified using the RSCS, compared to RNA-seq, and long-read data. PCA, principal component analysis; RSCS, RNA-seq combined strategy; TES, transcription end site; TSS, transcription start site.

In addition to the traditional RNA-seq, recently, long-read sequencing (GSE138760) on the Pacific Biosciences single-molecule real-time platform has been performed with early mouse embryos (39). To further evaluate the augmented transcriptome annotated using the RSCS, we performed a comparative analysis of the transcripts obtained by these strategies with the GENCODE annotation. First, we observed the length distribution of the RSCS transcripts was more similar to that of the annotated transcripts from GENCODE (Fig. 2*A*), showing the RSCS can generate a more complete transcriptome. Indeed, for instance, the pluripotency-associated gene *Nanog* was illustrated with Integrative Genomics Viewer, and the results showed that our strategy, but not RNA-seq and long-read sequencing, annotated all three transcript variants of *Nanog* in line with the GENCODE annotation, which were successfully validated by Sanger sequencing (Figs. 2*B* and S2, *A* and *B*). The programs assembling transcripts rely on three basic concepts: transcript structure, base composition, and sequence complexity (40, 41). We examined the number and length of exons in proportion from transcripts to assess the structure of transcripts, and the comparison shown that the RSCS transcripts displayed more similar characteristics to the GENCODE annotation than those from the RNA-seq and long-read sequencing (Fig. 2, *C* and *D*). In analysis of base composition, average percentage of each base in exon analysis revealed that the transcripts from the annotated transcriptomes showed no remarkable difference (Fig. 2*E*). However, further average percentage of motifs analysis suggested that the transcripts from the RSCS and RNA-seq, but not long-read data, were similar with the GENCODE annotation (Fig. 2*F*). We performed Markov model values in exons for complexity estimation. By principal component analysis (PCA), the RNA-seq annotated–transcripts were separated with the other annotations along PC1, and the RSCS transcripts were comparable with the GENCODE annotation (Fig. 2*G*). In addition, we also found the overlapping proportion of the exons of the RSCS transcripts with low-complexity regions obtained from RepeatMasker was in line with the transcripts annotated by long-read sequencing and GENCODE, but the transcripts from RNA-seq shown a bit much (Fig. 2*H*). The above results verified the reliability of our augmented transcriptome by the RSCS. To further address the accuracy of the novel transcripts annotated by the RSCS, we divided the transcripts defined in the mouse GENCODE into three parts and eliminated 20% transcripts in each part randomly, which were presumed as the unknown transcripts, and then we performed transcript annotation using the RSCS, RNA-seq, and long-read sequencing, respectively. Surprisingly, we found the TSS and transcription end site (TES) localizations of the eliminated transcripts identified by the RSCS were most matched with those from the GENCODE annotation, exhibiting high reproducibility in the three replicates (Figs. 2*I* and S2*C*). Altogether, we demonstrate that the RSCS can generate more complete and precise transcriptomes.

### Full-length transcripts annotated by the RSCS

**Figure 3:**
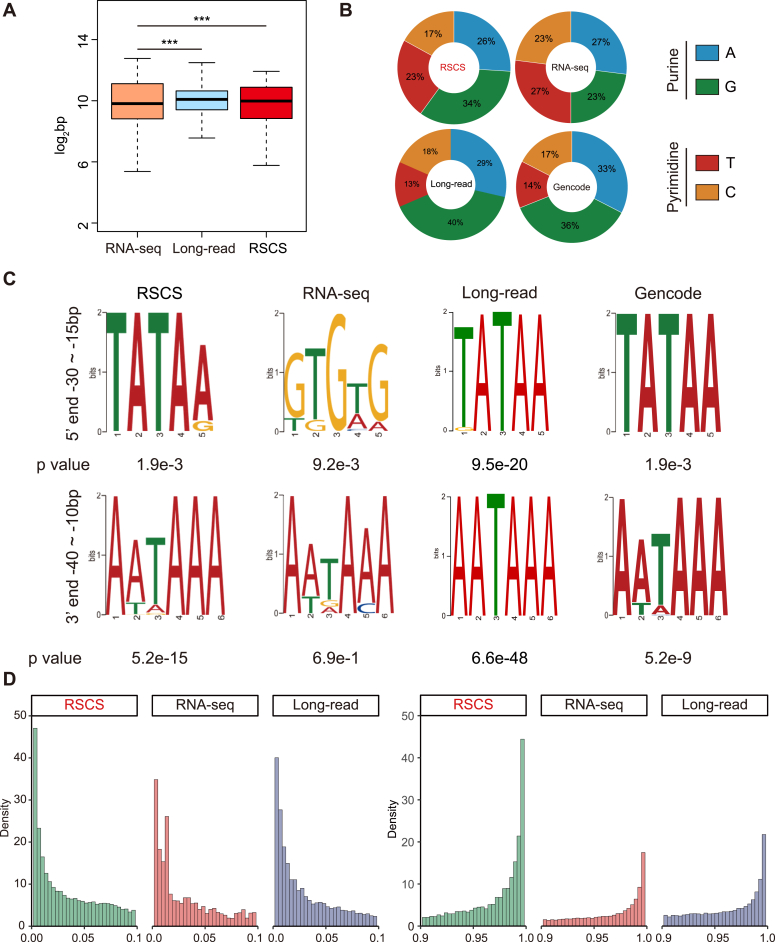
**Full-length transcripts annotation using the RSCS in mouse early embryos.***A*, *box plots* comparing the length of the transcripts annotated by the RSCS and those annotated using only RNA-seq data. ∗∗∗*p* < 0.001, two-sided *t* test. *B*, *pie chart* showing the percentages of the initial base of the transcripts identified by RSCS and those obtained based only on RNA-seq data. The percentages of each base are shown. *C*, frequently observed sequence motifs upstream of the initial and end bases of the transcripts. The region used for motif identification and the *p* value is shown. *D*, Kernel density estimates of the distribution of the TSS (*left*) and TES (*right*) locations of transcripts obtained using RSCS, RNA-seq, and long-read sequencing relative to the terminals defined in the mouse GENCODE (M26). RSCS, RNA-seq combined strategy; TES, transcription end site; TSS, transcription start site.

Above results shown that small RNAs exhibited a large coverage peak at the terminals of the RSCS transcripts, indicating small RNA reads might aid the annotation of full-length transcripts. To determine the integrity of the RSCS transcripts, we first compared the length of the RSCS transcripts with that of the transcripts annotated by RNA-seq and long-read sequencing, and the results showed that RSCS and long-read transcription was significantly longer than RNA-seq (Fig. 3*A*). In eukaryotic cells, RNA polymerases preferentially initiate transcription with a purine residue (42, 43). We observed that the initial bases of 60% of the transcripts identified using the RSCS were A (26%) and G (34%), resembling the profile of the GENCODE transcripts. However, the distribution of the initial bases of the transcripts obtained only based on RNA-seq was average (Fig. 3*B*). Then, we performed motif searching of core promoter and polyadenylation signal sequences upstream of the initial and end bases of the transcripts. In line with the GENCODE transcripts, the analysis of the RSCS and long-read sequencing transcripts showed clear TATAA and AATAAA motifs (Fig. 3*C*). In comparison, a nonenriched core promoter motif was obtained from the transcripts annotated by RNA-seq, and the polyadenylation signal analysis yielded a less significant *p* value (5.2e-15 *versus* 6.9e-1). We further examined the distribution of the TSS and TES locations of the transcripts obtained by each data, and this comparison shown that the most transcripts annotated by the RSCS could reach to the terminals defined by the GENCODE (Fig. 3*D*). Taken together, these data demonstrate that our newly developed method yielded full-length transcripts.

### Characterization of ERV-lncRNAs in mouse early embryos

**Figure 4:**
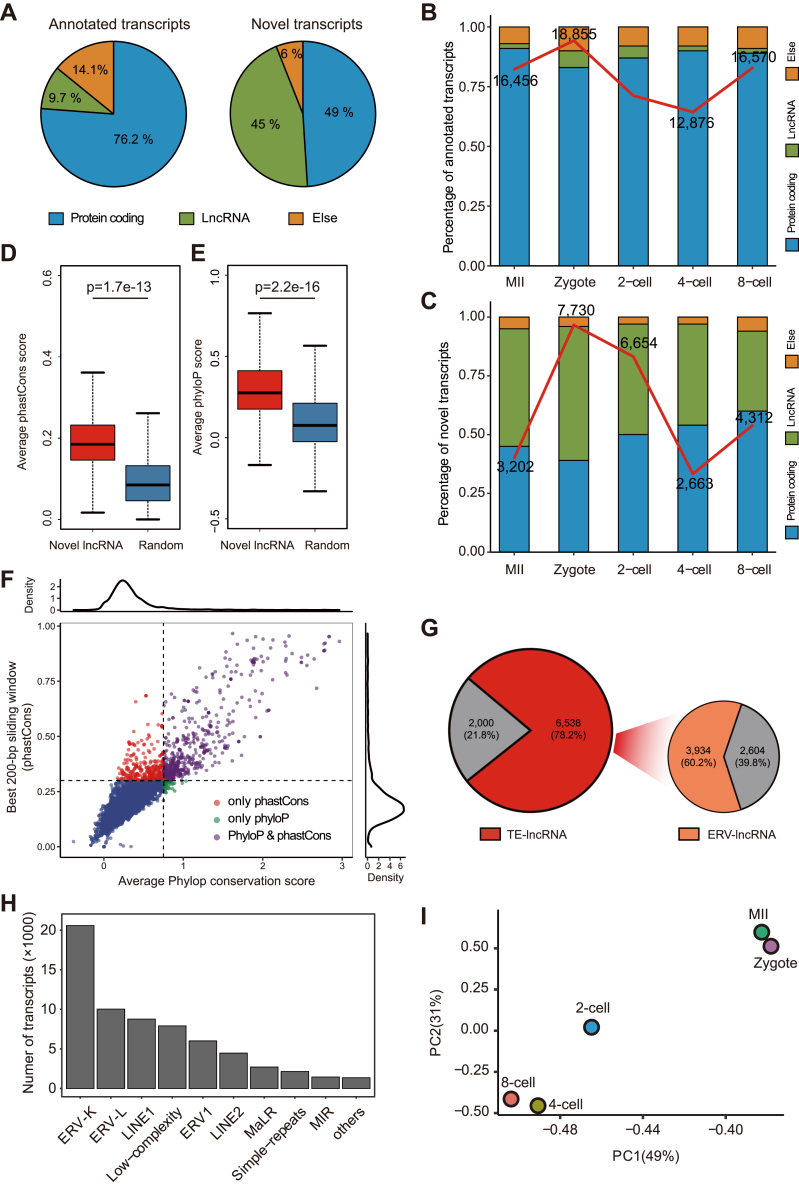
**Characterization of ERV-lncRNAs in mouse early embryos.***A*, classification of the annotated (*left*) and novel (*right*) RSCS transcripts according to the GENCODE annotation or protein-coding potential. *B* and *C*, classification of the annotated (*B*) and novel (*C*) transcripts in the stages according to GENCODE annotation. *Bar plot* represents the percentage of transcripts in each category and the *red line* represents the total number of the transcripts in each stage. *D* and *E*, comparison of the average phastCons score (*D*) and phyloP score (*E*) of the novel noncoding transcripts with sequences in random control regions. *p* values are calculated from two-tailed Wilcoxon rank sum tests. *F*, the *scatter plot* shows the conserved bases (the average phyloP conservation score >0.75) (*x*-axis) and the maximal 200-bp window average phastCons score (*y*-axis) of novel noncoding transcripts. *G*, Pie charts (*left*) showing the percentage of TE-associated lncRNAs in the early mouse embryos. And the TE-associated lncRNAs were classified to ERV-associated lncRNAs and and non ERV-lncRNAs (*right*). The analysis was based on the RepeatMasker database. *H*, enrichment of ERV subfamilies ordered by frequency. The frequency was calculated based on the counts of ERV subfamilies corresponding to the ranked expression of ERV-lncRNAs across all the stages. *I*, PCA of the ERV-lncRNA expression estimates in early mouse embryos. The expression of ERV-lncRNAs is characteristic of the different stages. EVR, endogenous retrovirus; LncRNA, long noncoding RNA; PCA, principal component analysis; RSCS, RNA-seq combined strategy; TE, transposable element.

Next, the RSCS transcripts, including the annotated transcripts and the novel transcripts, were further classified into protein coding transcripts and lncRNAs, according to the GENCODE annotation and protein-coding potential of the novel transcripts using CPAT (v2.2.0) (44) and CPC2 (v2.0) (45). We found that the majority of the annotated transcripts (15,731, 76.2%) were protein-coding transcripts and only 2003 transcripts (9.7%) were lncRNAs. In contrast, nearly half of the novel transcripts (6,366, 45%) were classified as lncRNAs (Fig. 4*A*). Moreover, in the annotated transcripts, the proportion of coding transcripts was over 80% in each stage (Fig. 4*B*), and in contrast, the novel lncRNAs accounted for majority of the novel transcripts (Fig. 4*C*). To evaluate the reliability of these novel lncRNAs, we performed sequence conservation analysis, according to both phyloP (46) and phastCons (47) scores. In general, the level of sequence conservation for the novel lncRNAs was significantly higher than that of random control regions (Fig. 4, *D* and *E*). Specifically, 760 transcripts among the novel noncoding transcripts showed higher conservation scores than those calculated from random control regions (phyloP and/or phastCons) (Fig. 4*F*), indicating the conservation of our newly identified lncRNAs. Abundance of lncRNAs, implicated in transcriptional regulation, have been evidenced to derive from TEs (48). A large number of TE-associated lncRNAs (78.2%) were identified from the RSCS lncRNAs and 60.2% of which were located in ERV elements (Fig. 4*G*). These ERV-lncRNAs were strongly associated with the ERVK subfamilies (Fig. 4*H*). The comparison of the ERV-lncRNAs to known coding transcripts showed that these lncRNAs tended to be weakly expressed and presented shorter lengths (Fig. S3, *A* and *B*). And, PCA of these ERV-lncRNAs clearly separated the two-cell embryo from the embryos at the other stages (Fig. 4*I*), revealing the specificity of ERV-lncRNAs from the two-cell embryo. Zygotic genome activation occurs at the two-cell stage in mice and is essential for the further development (49). Thus, we believe that the ERV-lncRNAs derived from the two-cell embryo might contribute to early embryonic development.

### Identification of novel functional ERV-lncRNA in early embryogenesis

**Figure 5:**
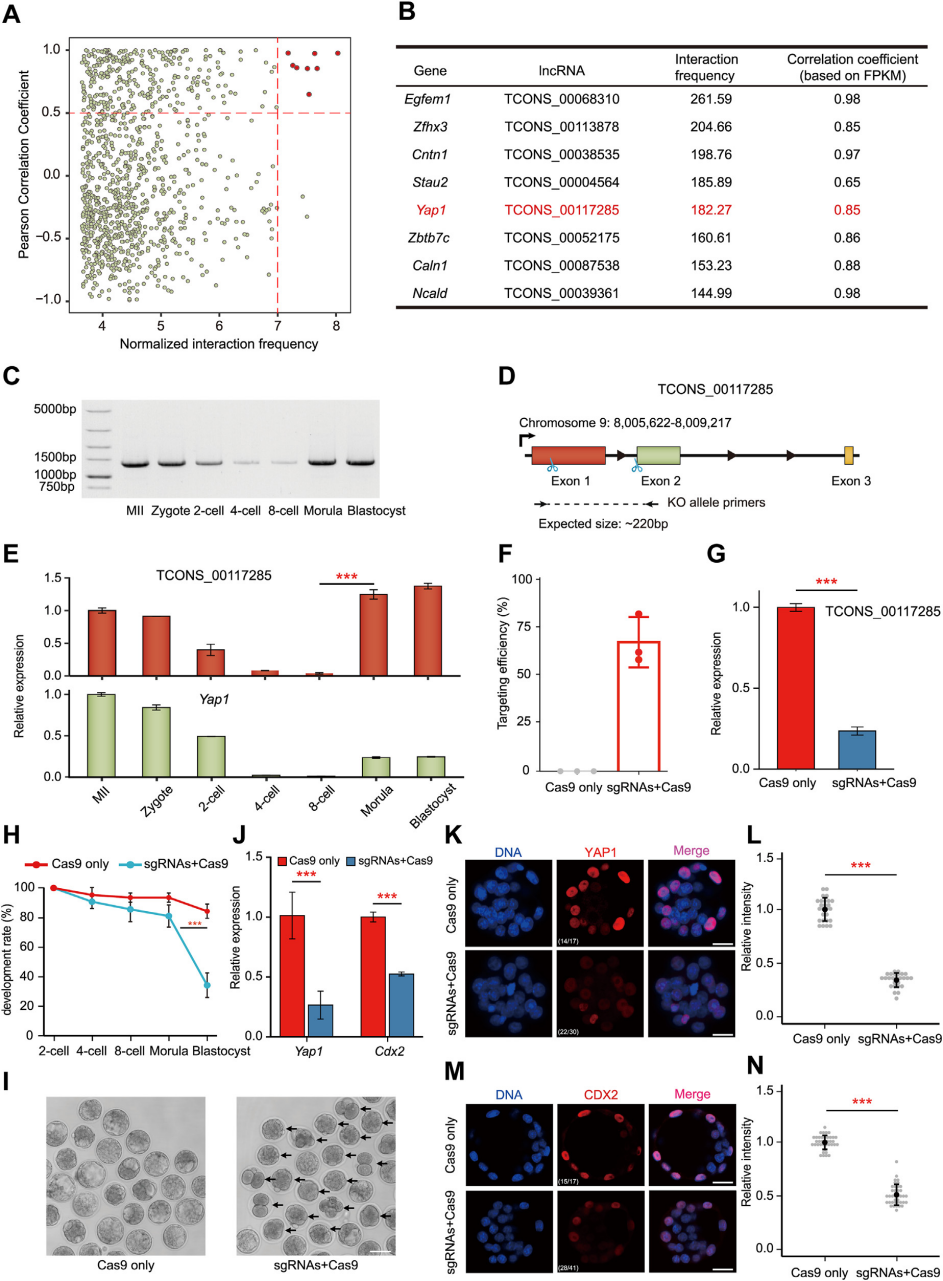
**TCONS_00117285 is involved in *Yap1* expression and is crucial for normal embryonic development.***A*, the *scatter plot* shows the normalized interaction frequency (*x*-axis) and the Pearson correlation coefficient (*y*-axis) of mRNA-lncRNA pairs in two-cell embryo. *B*, list of mRNA-lncRNA pairs highlighted in Figure 5*A*. The normalized interaction frequency and Pearson correlation coefficient are presented. *C*, RT-PCR validation of the full length of TCONS_00117285 during the early embryonic development. *D*, diagram illustrating the CRISPR-mediated KO of TCONS_00117285; the *blue scissors* indicate the loci of the guide RNAs. The *black arrows* represent the primers used for genotyping of the KO embryos. *E*, the relative expression of TCONS_00117285 and *Yap1* was measured by qRT-PCR analysis. Error bars indicate the SD. *F*, the targeting efficiency of TCONS_00117285 KO embryos were confirmed by single-embryo genotyping. The primers used for genotyping are included in Table S4. *G*, qRT-PCR analysis of the KO embryos revealing that the expression level of TCONS_00117285 was perturbed at the blastocyst stage. *H*, depletion of TCONS_00117285 impaired the development of preimplantation embryos. The sgRNAs and Cas9 mRNA mixture was injected into zygotes, followed by *in vitro* development. ∗∗∗ denotes *p* < 0.001, two-sided *t* test. *I*, morphological imaging of TCONS_00117285 KO embryos. The *arrows* indicate the abnormal embryos. The scale bars represent 100 μm. *J*, effect of TCONS_00117285 KO on the expressions of *Yap1* and *Cdx2*. Error bars indicate the SD. *K* and *M*, representative images of TCONS_00117285 KO embryos stained with YAP1 (*K*) and CDX2 (*M*). Figures at *bottom left* indicate the number of embryos exhibiting the morphology shown. The scale bar represents 20 μm. *L* and *N*, quantification of the YAP1 (*L*) and CDX2 (*N*) signal intensity. The average signal intensity of Cas9 only group was set as 1. Each *gray dot* represents a single embryo analyzed. *Center dot* and error bars indicate mean and SD., respectively. ∗∗∗ denotes *p* < 0.001, two-sided *t* test. Cas 9, CRISPR-associated protein 9; LncRNA, long noncoding RNA; RSCS, RNA-seq combined strategy; sg-RNA, single-guide RNA.

The mRNA-lncRNA pairs localized within the same topologically associated domain shows the highest correlation. To further characterize the ERV-lncRNAs that function in early embryogenesis, we analyzed the public high-resolution Hi-C data (GSE82185) of mouse two-cell embryo, combining with the expression correlation, and eight mRNA-lncRNA pairs maybe with the high correlation were found (Fig. 5*A*). We observed *Yap1*, a key transcriptional regulator for the first cell fate decision, and a novel identified ERV-lncRNA, TCONS_00117285, exhibited a significantly close connection (Pearson correlation coefficient >0.5, log_2_ (normalized interaction frequency) >7; Fig. 5*B*). To explore the role of TCONS_00117285, We first performed RT-PCR to amplify full-length of the transcript according to our RSCS annotation. RT-PCR and Sanger sequencing successfully validated its full length (Fig. 5, *C* and *D*). TCONS_00117285 transcribed from upstream of *Yap1* in an opposite direction, and there is no overlap with *Yap1* (Fig. S4*A*). Intriguingly, expression pattern of TCONS_00117285 and *Yap1* exhibited a highly expression correlation, and the expression of both of them were high at the morula and blastocyst stages (Fig. 5*E*). To test the function of TCONS_00117285, we constructed a pair of single-guide RNAs (sgRNAs) targeting TCONS_00117285 (Fig. 5*D*), and they were injected into zygotes with CRISPR-associated protein 9 (Cas9) mRNAs to perform TCONS_00117285 KO, followed by *in vitro* development. Genotyping of the blastocysts revealed that 67% of blastocysts carried genomic fragment deletions (Figs. 5*F* and S4*B*), and TCONS_00117285 exhibit significantly lower expression in sgRNAs/Cas9 blastocysts (Fig. 5*G*). Compared with the control group (only Cas9 injected), the efficient KO of TCONS_00117285 by using the CRISPR/Cas9 system resulted in abnormal embryo development at the morula and blastocyst stages (Fig. S5, *H* and *I*), suggesting TCONS_00117285 may function on ICM and trophectoderm specification. The specification of ICM and trophectoderm cells is dependent on YAP1 in the polarized outer cells, which translocate to the nucleus to activate trophectoderm-specific transcriptional factors (50). However, the mechanism of *Yap1* highly expressed in the outer cells has not been addressed. The highest interactions between *Yap1* and TCONS_00117285 was observed at the eight-cell stage before the formation of ICM and trophectoderm cells (Fig. S4*C*), indicating the role of TCONS_00117285 on the regulation of *Yap1* expression during the process of ICM and trophectoderm specification. As expected, we found that the level of *Yap1* was significantly reduced upon depletion of TCONS_00117285 (Fig. 5*J*). This observation was confirmed by immunofluorescence of YAP1 in the TCONS_00117285-KO blastocysts (Fig. 5, *K* and *L*). And, the more severe phenotype resulting from TCONS_00117285 depletion showed that the expression pattern and level of *Cdx2*, an important TE-specific transcriptional factor at the downstream of *Yap1*, were significantly affected at blastocyst stage checked by immunofluorescence (Fig. 5, *J*, *M* and *N*). In addition, the knockdown of TCONS_00117285 by RNAi also compromised the early embryonic development and attenuated the activation of *Yap1* and *Cdx2*, validate the importance of TCONS_00117285 on the regulation of *Yap1* (Fig. S4, *D*–*F*). Taken together, we identified a novel ERV-lncRNA that was functionally involved in control of *Yap1* expression and essential for early embryogenesis.

### Characterization of the RSCS transcripts during iPSC reprogramming

**Figure 6:**
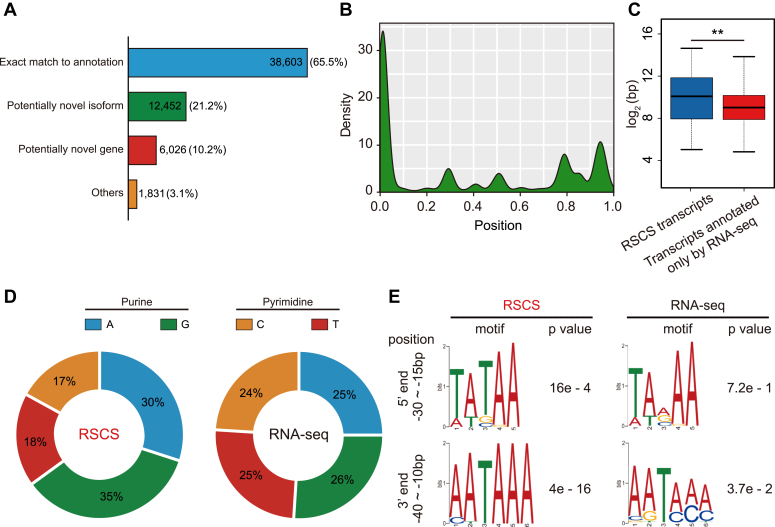
**Full-length transcripts in iPSC reprogramming annotated using the RSCS.***A*, annotation of the RSCS transcripts that were the combination of transcripts identified during iPSC reprogramming. The numbers and percentages of RSCS transcripts are presented. *B*, meta profile of small RNAs with respect to normalized full-length protein-coding genic regions. The coverage of small RNAs at the terminals was enriched, particularly at the 5′ ends. *C*, *box plots* showing the comparison of the length of the transcripts annotated using the RSCS and those obtained based on RNA-seq data. ∗∗*p* < 0.01, two-sided *t* test. *D*, *Pie chart* showing the percentages of the initial base of the transcripts identified by the RSCS and those obtained based on RNA-seq data. The percentages of each base are shown. *E*, frequently observed sequence motifs upstream of the initial and end bases of the transcripts. The region used for motif identification and the *p* values are shown. RSCS, RNA-seq combined strategy.

To test the credibility of RSCS for transcriptome annotation in other cellular contexts, we extended the strategy to induced pluripotent stem cell (iPSC) reprogramming using public RNA-seq and small RNA-seq data (GSE102518). We annotated 58,912 transcripts using the RSCS (Fig. 6*A*, Tables S1 and S3). Also, the small RNA tags were positioned at the transcript terminals (Fig. 6*B*). Importantly, the RSCS transcripts in iPSC reprogramming shared key characteristics with those found in mouse embryos, showing longer length (Fig. 6*C*), preferred initial purine (Fig. 6*D*), and clearly terminal motifs (Fig. 6*E*). Taken together, these results demonstrate that our strategy can annotate full-length transcripts in a wide variety of cellular contexts.

## Discussion

The pervasive transcription of mammalian genomes into various long, short, protein-coding, and noncoding transcript classes has been evidenced by large-scale studies (51). These projects emphasize the need for complementary high-throughput technologies coupled with integrative bioinformatic approaches to characterize the diversity of RNA transcripts. Here, we developed a computational pipeline that integrates RNA-seq and small RNA-seq data, denoted RSCS, and this strategy greatly improves the resolution and accuracy of transcriptome annotation in a wide variety of mammalian samples. We characterized the deepest part of the transcriptome, which is essential for understanding the complexity of previously unannotated RNAs, with potential regulatory features, such as the ERV-lncRNAs described in this study.

RNA-seq has greatly facilitated the annotation of functional, physiological, and biosynthetic cellular states at the organismal, tissue, and even single-cell levels. However, due to the limited coverage of the short reads produced by a low quantity of RNA, full-length transcripts are difficult to obtain using the traditional method (20). Long-read sequencing technology has been widely used for annotation and can overcome these limitations, but it fails to profile short transcripts. In addition, sufficient complementary DNAs are needed for long-read sequencing to avoid size selection and bias generation (52). Therefore, optimized analysis methods are desired for the construction of a more complete transcriptome. Small RNAs derived from coding transcripts, particularly the 5′ and 3′ UTRs, have been observed, and the phenomenon indicates that small RNA reads might aid the annotation of transcripts. As evidenced by the length comparison, the frequency of the core promoter and the polyadenylation signal motifs, and the locations of TSS and TES, the transcripts annotated with our strategy appear to be full length. Overall, the boundaries and length distribution of the transcripts identified by the RSCS was most consistent with the reference annotation, which suggests that the identified transcripts are a genuine representation of the expressed transcript set. Additionally, although this finding needs to be further validated, thousands of high-confidence potential novel transcripts were identified in various cellular contents, and these identified transcripts provide a valuable resource for clarifying the molecular basis of the specific biological events. The results demonstrate that the RSCS can generate a more complete and precise transcriptome.

Many unusual transcripts, such as those from repeat elements, are excluded by bioinformatic analysis due to their low expression. In this study, TE-associated lncRNAs were identified by the RSCS and a large fraction of these were ERV-lncRNAs. Consistent with the above-described observation that small RNAs were positioned at the terminals of transcripts, we found that the small RNA reads could help annotate the transcripts, which showed the clear advantage of the RSCS for exploring novel transcripts, particularly those found at low abundances. ERVs are a major source of genetic innovation and can generate specific regulatory sites of nearby genes (13). Because ERVs have expanded chromatin-binding sites for some transcription factors, the transcripts derived from their locus, such as the ERV-lncRNAs described in the study, show highly stage-specific expression, which indicates that the transcripts associated with the distinct ERV subfamilies contribute in different ways to the transcriptome. In addition, an ERV-lncRNA was identified to function on early embryogenesis. Importantly, our strategy generates a variety of stage-specific ERV-lncRNAs, and only a subset of them we have characterized have been studied thus far in other contexts. Therefore, our analysis highlights the unexplored potential of ERV-lncRNAs for tagging, characterizing, maintaining, or inducing cellular identity.

In summary, we applied the RSCS to annotate augmented but high-confidence potential transcriptomes with more complete and precise contents, and taking advantage of our strategy, we identified a large number of ERV-lncRNAs, which indicates that the set of functions of retrotransposons in specific biological events has only just begun to be unraveled.

## Experimental procedures

### Mouse experiments

Animal welfare and the experimental procedures adhered to the ethical provisions on the Care and Use of Experimental Animals of Wenzhou Medical University and were approved and authorized by the Animal Experimental Committee of the University (No. xmsq2021-0068). Animal experiments were performed with 7-week-old ICR mice. Animals were maintained under a 12 h light/dark cycle and provided with food and water ad libitum in individually ventilated units.

### Embryos collection

Embryos were collected from 7-week-old F1 superovulated female mice treated with 6.5 international unit of pregnant mares’ serum gonadotropin and, 45 to 47 h later, with 5 IU of human chorionic gonadotropin and crossed to ICR males. Embryos were isolated in M2 medium (Sigma: M7167) and cultured in potassium-supplemented optimised medium (Merck: MR-121-D) at 37 °C in 5% CO2. Zygotes stage embryos were treated with hyaluronidase to remove cumulus cells.

### Microinjection

All the sgRNAs used in this study were purchased from GenScript. sgRNA-204: 5′-TTATCTATCAGGAGCCTATC-3′; sgRNA-1085: 5′-CCAGTTTGTGGCACAAGGCG-3′. The mixtures containing two sgRNAs (50 ng/μl) and Cas9 (100 ng/μl; Invitrogen, A29378) were injected into the cytoplasm of zygotes as described as follows. The Cas9 mRNAs as negative control. In RNAi experiment, siRNAs were purchased from GenScript. si-7285 1s: UGUUUUGAGUUGAAGUUCCCA; si-7285 1as: GGAACUUCAACUCAAAACAUA; si-7285 2s: UUGUUUGUGGAUUAACCGCUG; si-7285 2as: GCGGUUAAUCCACAAACAAAA; si-7285 3s: AUUAGUCUGUGAACCAAUCCU; si-7285 3as: GAUUGGUUCACAGACUAAUUA; To perform microinjection, zygote-stage embryos were placed in 150 μg/ml hyaluronidase (Sigma) to digest the outer granule cells. The RNA mixtures were centrifuged at 12,000 rpm for 10 min at 4 °C and placed at 4 °C for use. Then, microinjection was carried out with an Eppendorf FemtoJet 4i microinjector and Nikon E LIPSE Ti micromanipulators. For injection, a glass capillary Femtotip (Eppendorf) was loaded with 2 μl of RNA mixtures by a microloader (Eppendorf), and the solution was injected into the cytoplasm in a drop of M2 medium. The injection volume was approximately 2 to 5 pL. The injection conditions consisted of 250 hPa injection pressure, 60 hPa compensation pressure, and 0.7 s injection time. Immediately after microinjection, embryos were cultured in KSOM medium at 37 °C in 5% CO2.

### Embryo lysis and genotyping

Each individual knockout embryo was transferred to PCR tubes containing 5 μl lysis buffer (50 mM Tris–HCl (pH 8), 400 μg/ml Proteinase K, 0.5% Triton 20). Embryo lysis was performed at 60 °C for 1 h, following heat inactivation at 90 °C for 5 min and 2 μl of lysis buffer containing genomic DNA was used as template for nested PCR. The target region was PCR amplified with Phusion Green Hot Start II High-Fidelity PCR Master Mix (Thermo Fisher Scientific). The primers used for genotyping are included in Table S4 (WT allele 1101 bp and KO allele ∼220 bp). For first round of PCR, the following program was used: initial denaturation, 30 s at 98 °C, 30 cycles of 10 s at 98 °C, and 40 s at 72 °C and final extension, 10 min at 72 °C. For second round of PCR, the following program was used: initial denaturation, 30 s at 98 °C, 30 cycles of 10 s at 98 °C, 30 s at 60 °C, and 4 s at 72 °C and final extension, 10 min at 72 °C.

### Immunofluorescence staining

After removal of the zona pellucida with acidic operating fluid, mouse embryos were fixed in fixative Solution (Invitrogen, cat.FB002) for 40 min at room temperature (RT), followed by permeabilization in 1% Triton X-100 for 20 min at RT. Embryos were then blocked in blocking solution (1% BSA in PBS) for 1 h at RT after three washes in washing solution (0.1% Tween-20, 0.01% Triton X-100 in PBS). Antibodies incubations (YAP1: CST, 14074; CDX2: Abcam, cat.76541) were performed overnight at 4 °C. The next day, the embryos were washed in washing solution and incubated with secondary antibodies for 1 h at RT. After staining with Hoechst, the embryos were washed in washing solution. Imaging of embryos was performed using an inverted confocal microscope (Leica TCS SP8).

### RNA extraction, reverse transcription, and q-PCR analysis

Total RNA was extracted using the Arcturus PicoPure RNA Isolation Kit (Ambion) according to the manufacturer’s instructions, and reverse transcription was performed to generate cDNA using the PrimeScript RT Master Mix (Takara). RNase-Free DNase Set (QIAGEN) was used to ensure that there was no DNA contamination. Q-PCR was performed using the TB Green Premix Ex Taq II (Takara) and CFX96 Real-Time System (Bio-Rad). The reaction parameters were as follows: 95 °C for 30 s followed by 40 two-step cycles of 95 °C for 5 s and 60 °C for 34 s. *Hprt* were used as reference genes. Ct values were calculated using System software (Bio-Rad), and the amount of target sequence normalized to the reference sequence was calculated as 2^−△△Ct^.

### The RSCS pipeline

The RSCS pipeline includes: (i) preconfiguration of sample data; (ii) modified transcriptome assembly; and (iii) transcripts classification and lncRNA prediction.

#### Preconfiguration of sample data

The raw sequence data in FASTQ format were filtered to remove reads with unknown nucleotides, and FastQC (v0.11.5) was used for Illumina reads. Subsequently, Trim Galore (v0.6.4) software (https://www.bioinformatics.babraham.ac.uk/projects/trim_galore/) was used to discard low-quality reads, trim the adaptor sequences, and eliminate poor-quality bases with parameter -q 30. Specifically, it is worth noting that the trimmed reads from the small RNA-seq data with less than 18 nts or more than 50 nts were discarded by Trim Galore (parameter: “--small_rna --length 18”). To minimize the batch effect, RNA-seq and small RNA-seq at each stage of preimplantation embryo were merged with the equal number of reads. RNA-seq and small RNA reads were mapped to mm10 using HISAT2 (v2.1.0) and the unique reads were used in further analyses.

#### Modified transcriptome assembly

We then transformed sequence alignment map (SAM) format files to Binary Alignment Map (compressed binary version of the SAM format) format, discarded reads that were not aligned to the reference genome, and sorted the BAM file with SAMtools (v1.9).

For deep annotation, we considered the following features:

• BAM files are generated after alignment and after mapped reads with a low quality have been filtered out.
• BAM is the compressed binary version of the SAM format, uses Blocked GNU Zip Format compression and can support indecies to achieve fast random access by generating BAM index files.

The BAM files of RNA-seq and small RNA-seq data obtained at identical biological stages were merged using SAMtools merge, and a pooled BAM file was generated. Importantly, only reads with MAPQ values over 10 and that mapped to a single locus in the genomes were used in this study. These steps were conveniently performed simultaneously with the duplicate marking by running MarkDuplicates on all read groups in each pooled BAM file obtained for a sample, and this procedure was repeated for each sample. We then ran base recalibration on the aggregated per-sample BAM files.

Transcript assemblies were performed for each sample separately using StringTie (v1.2.3) (53) with GENCODE annotation (vM20) (54) as a guide. The expression level of each gene was quantified by normalized FPKM using StringTie (v1.2.3). For new transcripts, data normalization was performed by transforming summational mapped transcript reads to RPKM (Reads Per Kilobase per Million mapped reads).

#### Transcripts classification and lncRNA prediction

We used StringTie merge to merge all the transcripts identified by the RSCS across all stages. The transcripts were annotated through a comparison with mouse GENCODE annotation (vM20) using Cuffcompare (v2.2.1) and classified the long-read transcripts into four classes according to their most closely matching GENCODE transcript. The set of “=” for “exact match to annotation,” the set of “e” and “j” for “potentially novel isoform,” “u” for “potentially novel gene,” and the other codes for “other”.

To identify reliable, multiexonic long noncoding transcripts, we implemented the following selection criteria: (i) read coverage ≥3 in at least one of the tissues; (ii) ≥200 bp; (iii) ≥2 exons; (iv) low protein–coding potential predicted by CPC2 and CPAT; and (v) no sense overlap with known coding genes derived from UCSC, Ensembl, and Refseq databases.

### Annotation of ERV-lncRNAs

Coordinates and annotations of TE elements were downloaded from the UCSC Genome Browser (7/3/2012 version of RepeatMasker). LncRNAs overlapping with TE elements were identified as TE-lncRNAs using BEDTools software (https://github.com/arq5x/bedtools2). ERV-lncRNAs were further identified by RepeatMasker annotation. The ERV-lncRNAs with an expression level (FPKM) >1 were used for further analysis. ERV-lncRNA PCA plot were performed in the R language.

### ChIP-seq data processing

The gene read densities were generated using deepTools computeMatrix. To analyze the enrichment signal in super enhancers, we used a predefined genome-wide super enhancer annotation obtained from a previous study as the reference regions (55). Chromatin immunoprecipitation followed by sequencing (ChIP-seq) reads (GSE102518) that are aligned to the regions were extended by 200 bps, and the density of reads per base pair was calculated. The density of reads in each region was normalized to the total number of million mapped reads to obtain a read density in units of reads per million mapped reads per base pair (rpm/bp). The regions were sorted in descending order based on the mean value of the ChIP-seq signal per region and visualized using deepTools plotProfile.

### Hi-C analyses

To process Hi-C data, we first trimmed the raw data to remove adaptor sequences and low-quality reads. Paired-end raw reads of the Hi-C libraries were aligned, processed, and iteratively corrected using HiC-Pro (v2.11.4) (https://github.com/nservant/). Briefly, the sequencing reads were first independently aligned to the mouse reference genome (GRCm38.p6) using the bowtie2 end-to-end algorithm. Unmapped reads, multiple mapped reads, and singletons were then discarded, and valid read pairs were then binned at a specific resolution by dividing the genome into bins of equal size. We selected a bin size of 10 kb to show local interactions and to perform topologically associated domain calling.

### Identification of the mRNA-lncRNA pairs

To detect potential mRNA-lncRNA pairs in preimplantation embryos, Pearson's correlation coefficients between mRNA-lncRNA pairs of different RNA-Seq replicates were calculated in each stage of preimplantation embryo *via* corrr package in the R language. And, the binned interaction matrices of HiC data were normalized using the iterative correction method to correct for biases such as GC content, mappability, and effective fragment length in Hi-C data. To determine the interactions of mRNA-lncRNA pairs, the interaction scores in the region were calculated by Bedtools, and the average interaction frequencies within the defined genomic regions from different replicates were calculated.

### Statistical analysis

The statistical analyses were performed using R4.0.2 (https://cran.r-project.org/bin/windows/base/old/4.0.2/). The data are shown as the means ± SEMs. Differences between the results obtained for two groups were evaluated using either two-tailed Student’s *t* test or the Wilcoxon rank sum test. The asterisks indicate significant differences: ∗ *p* < 0.05, ∗∗ *p* < 0.01, and ∗∗∗ *p* < 0.001.

## Data availability

RNA-seq, small RNA-seq, long-read sequencing, and Hi-C data in mouse embryos were downloaded from previous publications (GSE70605, GSE83581, GSE138760, and GSE82185) (39, 56, 57, 58). RNA-seq, small RNA-seq, and ChIP-seq data were also downloaded from previous publications (GSE102518) (59).

## Code availability

The pipeline presented in this paper has been packaged in the RSCS. The source code and associated instructions can be downloaded from (https://github.com/summus-kong/RSCS).

## Supporting information

This article contains supporting information.

## Conflict of interest

The authors declare that they have no conflict of interest with the contents of this article.
