# Supplementary material for: Deep annotation of long noncoding RNAs by assembling RNA-seq and small RNA-seq data
Source: J Biol Chem. 2023 Aug 4;299(9):105130. doi: 10.1016/j.jbc.2023.105130 (PMC10498003; doi:10.1016/j.jbc.2023.105130)
Supplement: Supporting Figures S1–S4 and Tables S1–S4 [file mmc1.pdf]

Deep annotation of long noncoding RNAs by assembling RNA-seq and small RNA-seq data

Jiaming Zhang[1,2,3], Weibo Hou[1,3], Qi Zhao[2,3], Songling Xiao[1], Hongye Linghu[1], Lixin Zhang[1], Jiawei Du[1], Hongdi Cui[1], Xu Yang[1], Shukuan Ling[2,*], Jianzhong Su[2,*] and Qingran Kong[1,*]

[1]Oujiang Laboratory, Zhejiang Provincial Key Laboratory of Medical Genetics, Key Laboratory of Laboratory Medicine, Ministry of Education, School of Laboratory Medicine and lif e Sciences, Wenzhou Medical University, Wenzhou, Zhejiang, China. [2]Oujiang Laboratory, Zhejiang Lab for Regenerative Medicine, Vision and Brain Health, Wenzhou Medical University, Wenzhou, Zhejiang Province, China; [3]These authors contributed equally: Jiaming Zhang, Weibo Hou, Qi Zhao.

* Corresponding authors: kqr721726@163.com; sujz@wmu.edu.cn; sh2ling@126.com.

Supplementary Information:
Supplementary Figure S1-S4
Supplementary Table S1-S4

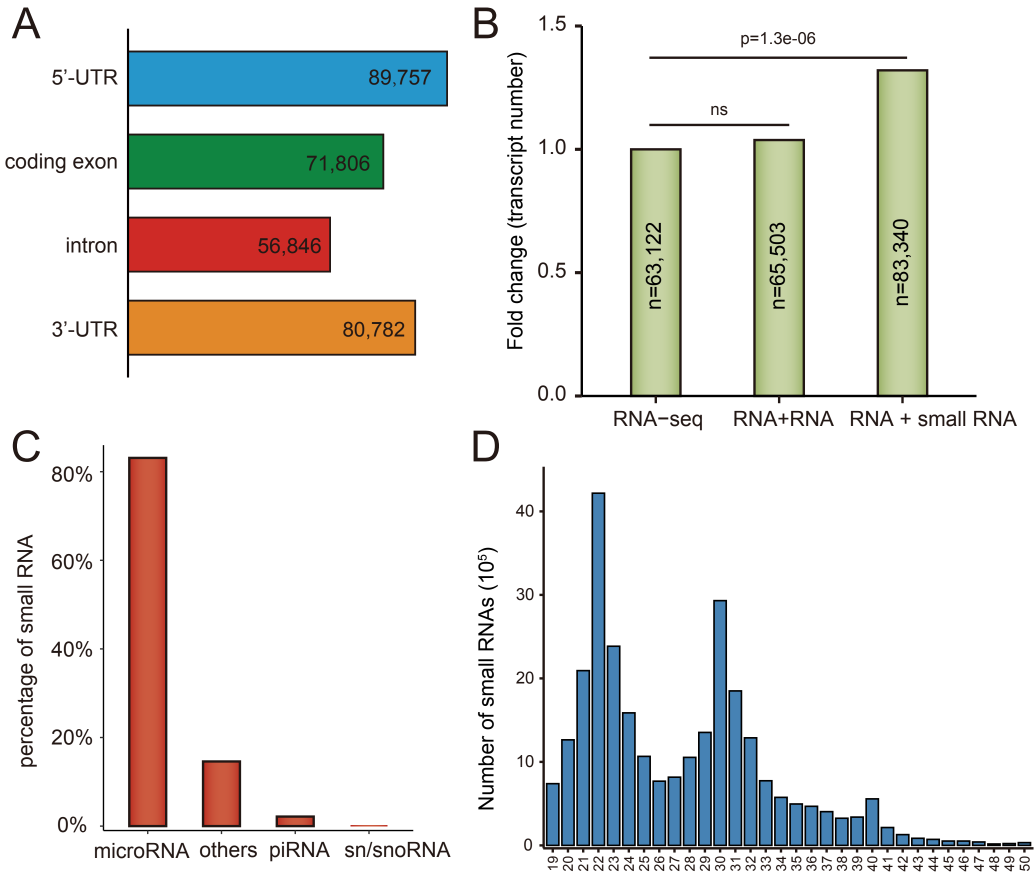
A
5'-UTR
89,757
coding exon
71,806
intron
56,846
3'-UTR
80,782
B
p=1.3e-06
ns
Fold change (transcript number)
1.5
1.0
0.5
0.0
n=63,122
n=65,503
n=83,340
RNA−seq
RNA+RNA
RNA + small RNA
C
percentage of small RNA
80%
60%
40%
20%
0%
microRNA
others
piRNA
sn/snoRNA
D
Number of small RNAs (10⁵)
40
30
20
10
0
19 20 21 22 23 24 25 26 27 28 29 30 31 32 33 34 35 36 37 38 39 40 41 42 43 44 45 46 47 48 49 50

Figure S1 Genome-wide distribution of small RNAs from mouse early embryos.

(A) Genome-wide distribution of small RNAs obtained from the combination of small RNAs identified at the five stages. The numbers of the merged RNAs belonging to the four categories are shown. (B) The number of transcripts annotated by RSCS and daul RNA-seq data. (C) Classification of small RNAs positioned to the 5’- and 3’-UTRs of the transcripts. Using publicly available databases (miRBase and piRNABank), the small RNAs identified at each stage were divided into the four indicated categories. (D) Terminal small RNAs exert the bimodal pattern distribution.

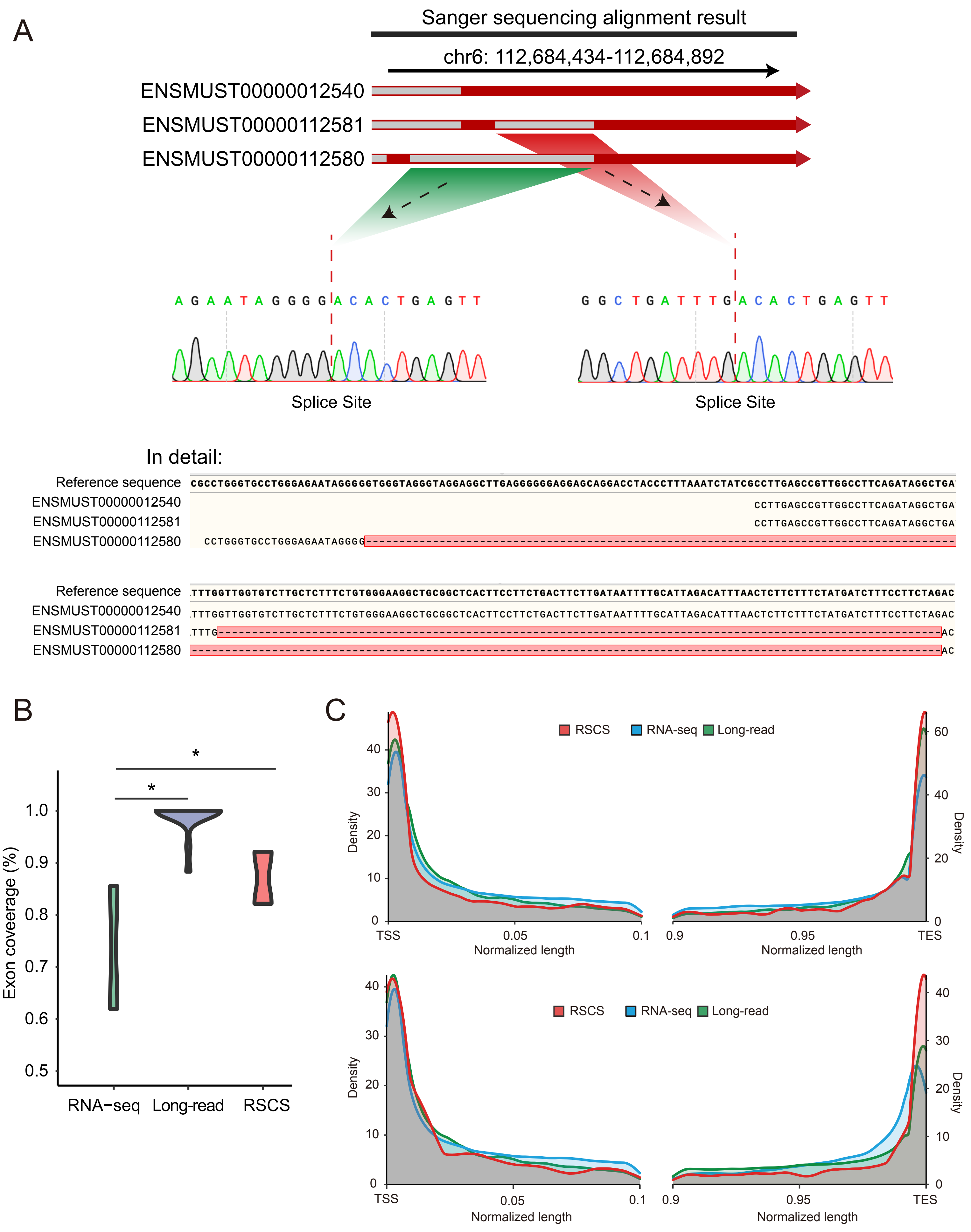
A
Sanger sequencing alignment result
chr6: 112,684,434-112,684,892
ENSMUST00000012540
ENSMUST00000112581
ENSMUST00000112580
A G A A T A G G G G A C A C T G A G T T
Splice Site
G G C T G A T T T G A C A C T G A G T T
Splice Site
In detail:
Reference sequence
ENSMUST00000012540
ENSMUST00000112581
ENSMUST00000112580
CCTGGGTGCCTGGGAGAATAGGGG
Reference sequence
ENSMUST00000012540
ENSMUST00000112581
ENSMUST00000112580
B
Exon coveerage (%)
1.0
0.9
0.8
0.7
0.6
0.5
*
*
RNA−seq
Long-read
RSCS
C
RSCS
RNA-seq
Long-read
Density
TSS
0.05
0.1
0.9
0.95
TES
Normalized length
Density

Figure S2 Validation of the RSCS transcripts.

(A) Sanger sequencing chromatograms of the RT-PCR products confirming all three transcript variants of Nanog were expressed in blastocyst. Sanger sequencing reads were aligned to reference sequence (chr6: 112,684,434-112,684,892, upper) by SnapGene. The splice sites were highlighted (middle). The details of the sequence alignment in SnapGene are shown below. (B) Box plots showing the bam coverage of Nanog transcripts annotated by the RSCS, RNA-seq and long-read data. (C) Kernel density estimates of the distribution of the TSS and TES localizations of the transcripts identified using RSCS, compared to RNA-seq and long-read data.

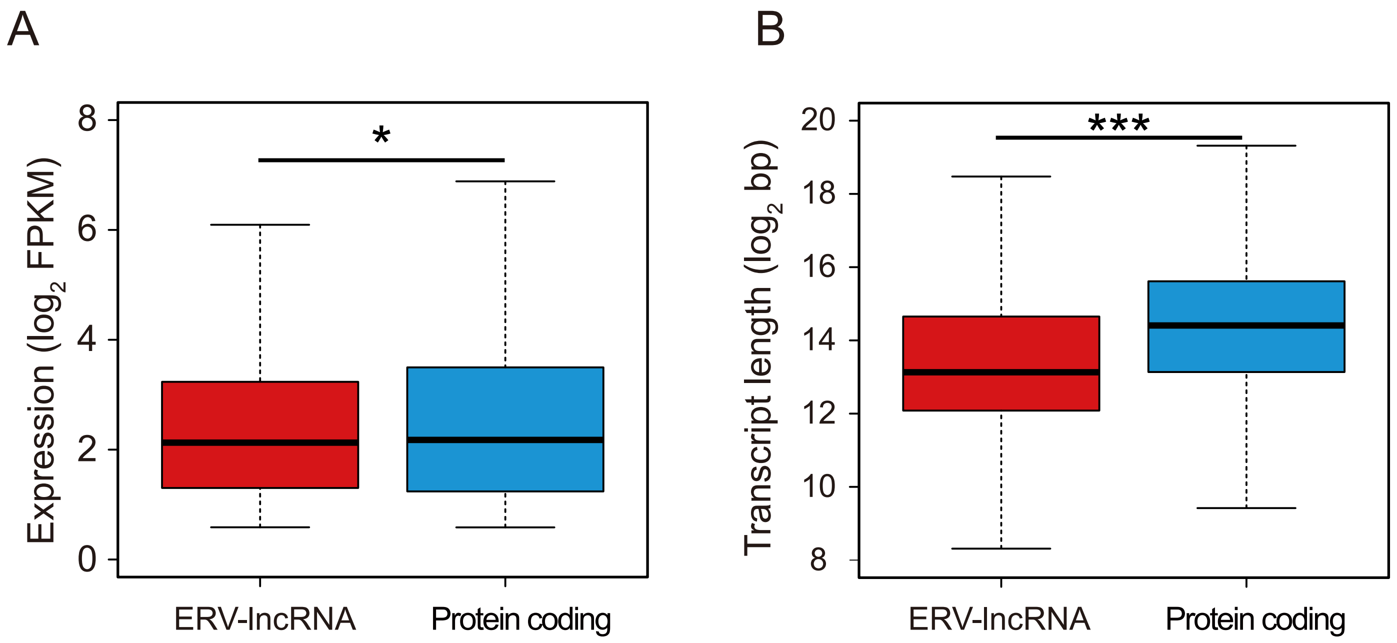
A
B
Expression (log2 FPKM)
0
2
4
6
8
*
ERV-lncRNA
Protein coding
Transcript length (log2 bp)
8
10
12
14
16
18
20
***
ERV-lncRNA
Protein coding

Figure S3 Comparison of expression and transcript length of ERV-lnRNAs.

(A, B) Normalized expression (A) and transcript length (B) of transcripts for ERV-lncRNAs, and coding transcripts obtained by the RSCS. *P < 0.05 from two-sided Student's t test are shown.

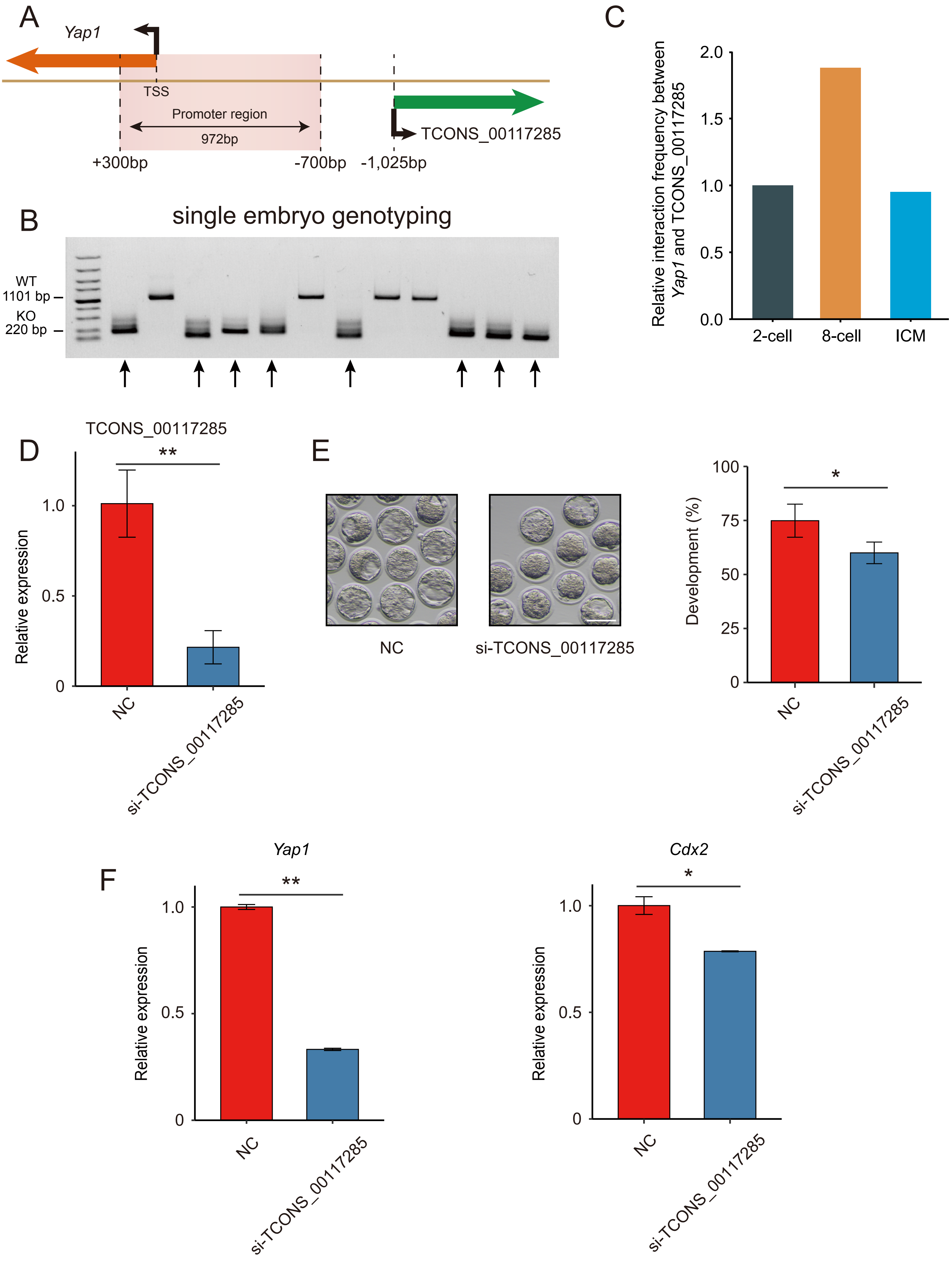

A
Yap1
TSS
Promoter region
972bp
TCONS_00117285
+300bp
-700bp
-1,025bp
B
single embryo genotyping
WT
1101 bp
KO
220 bp
C
Relative interaction frequency between Yap1 and TCONS_00117285
2.0
1.5
1.0
0.5
0.0
2-cell
8-cell
ICM
D
TCONS_00117285
**
Relative expression
1.0
0.5
0
NC
si-TCONS_00117285
E
NC
si-TCONS_00117285
Development (%)
100
75
50
25
0
*
NC
si-TCONS_00117285
F
Yap1
**
Relative expression
1.0
0.5
0
NC
si-TCONS_00117285
Cdx2
*
Relative expression
1.0
0.5
0
NC
si-TCONS_00117285

Figure S4 Deletion of TCONS_00117285 compromised the early embryonic development.

(A) Diagram illustrating the location and transcriptional direction of *Yap1* and TCONS_00117285. TCONS_00117285 transcribed in an antisense direction against *Yap1*. And, there is no overlap between TCONS_00117285 and *Yap1*. *Yap1* promoter, which spanned 972 bp and located at -700 bp to +300 bp of the TSS, identified by CDX2, HNF4 and H3K27ac ChIP data (PMID: 31216773). (B) An agarose gel image illustrating the nested PCR-based genotyping assay of the TCONS_00117285 knockout blastocysts. Genotyping results of 12 embryos are shown in this image. Black arrows point to the embryos carried genomic fragment deletions. (C) The bar plot showing the relative interactions between *Yap1* and TCONS_00117285 at different stages. (D) qPCR validating the TCONS_00117285 knockdown efficiency. (E) Representative images (left) and developmental rates (right) of control and TCONS_00117285 knockdown embryos. Scale bars, 100 μm. (F) Effect of TCONS_00117285 knockdown on the expressions of Yap1 and Cdx2. Error bars indicate the s.d.
